# Transcriptomics Identifies Differentially Expressed Genes Inducing Tuber Formation in Early- and Late-Maturing Potatoes

**DOI:** 10.3390/plants13131879

**Published:** 2024-07-08

**Authors:** Yongzhen Ma, Mengtao Li, Shujuan Wang, Ke Deng, Long Zhao, Jia Luo, Wenquan Wang, Fang Wang, Jian Wang

**Affiliations:** 1 Qinghai University, Xining 810016, China; myz19940714@sina.com (Y.M.); dengker87@163.com (K.D.); 2 Academy of Agriculture and Forestry Sciences, Qinghai University, Xining 810016, China; 3 National Key Laboratory of Sanjiangyuan Ecology and Plateau Agriculture and Animal Husbandry, Laboratory for Research and Utilization of Qinghai Tibet Academy of Agriculture and Forestry Sciences, Qinghai University, Xining 810016, China; 4 National Key Laboratory of Sanjiangyuan Ecology and Plateau Agriculture and Animal Husbandry, Laboratory for Research and Utilization of Qinghai Tibet Plateau Germplasm Resources, Qinghai University, Xining 810016, China; 5 Key Laboratory of Qinghai-Tibet Plateau Biotechnology Ministry of Education, Qinghai University, Xining 810016, China; 6 Qinghai Provincial Key Laboratory of Potato Breeding, Ministry of Education, Engineering Research Center of Potato in Northwest Region, Qinghai University, Xining 810016, China; 7 Institute of Tropical Agriculture and Forestry, Hainan University, Haikou 570228, China; qsea1573@163.com (M.L.); wangshujuan113@yeah.net (S.W.); luo19980309@foxmail.com (J.L.); wangwenquan@itbb.org.cn (W.W.); 8 Hainan Yazhou Bay Seed Laboratory, Sanya Nanfan Research Institute, College of Tropical Crops, Hainan University, Sanya 572025, China; longz0322@hainanu.edu.cn

**Keywords:** potato, grafting, transcriptome, *StYABBY1*

## Abstract

The timing of potato tuberization is affected by potato ripeness, environmental factors, and polygene regulation. The accurate control of the transition to tuberization has both scientific and practical production value, but the key factors regulating this transition remain unclear. This study grafted an early-maturing potato variety (Favorita) scion to the late-maturing Qingshu 9 variety and demonstrated that a heterologous early-maturing scion can induce early potato formation on a late-maturing rootstock. The transcriptome of functional leaves and stolons of grafted plants was comprehensively analyzed and 593 differentially expressed genes (DEGs) were identified, including 38 transcription factors. Based on gene molecular function analysis and previous reports, we propose that *PIF5*, *bHLH93*, *CBF3*, *ERF109*, *TCP19*, and *YABBY1* are the key DEGs that induce tuber formation in early- and late-maturing potatoes. The *YABBY1* gene was subjected to functional verification. The leaf area of *StYABBY1*-overexpressing plants was smaller than the wild type and no potato tubercles were formed, while an RNA interference plant line showed no change in leaf area and formed tubers, indicating that *StYABBY1* has a role in leaf size regulation and tuber formation.

## 1. Introduction

Potato (*Solanum tuberosum* L.) is the world’s fourth-largest staple crop after wheat, rice, and maize [1]. It is widely grown in over 150 countries and is a staple food for more than 1.3 billion people due to its adaptability, nutritional content, and low production costs [2]. Differences between potato varieties in flowering time, tuberization time, leaf senescence, and life cycle duration are the most important agronomic traits for distinguishing potato maturity [3]. However, the key factors regulating potato setting time remain unclear.

Grafting is an ancient agricultural production technique with a 4000-year history in China. It is widely used in research into scion material exchange and signaling between rootstocks [4,5,6]. The rootstock can control a plant’s structure by strengthening the scion, inducing early flowering, and enhancing pest and disease resistance and soil adaptability. RNA, proteins, hormones, and even chloroplasts and nuclear genomes can be transported from rootstock to scion [7,8,9]. The key genes for potato tuber formation (*StBEL5*, *StPOTH1*, and *StSP6A*) can be transferred downward to the stolon tip through the grafting junction to regulate tuber formation [10,11,12]. The miRNA172 molecule controls potato tuber formation and can be transported to stolons over long distances through grafted junctions, regulating the expression of the target tuber development gene StBEL5 and promoting tuber enlargement [13]. Long distance-transported miRNAs respond to various physiological processes arising from grafting and can regulate plant growth and development and response to environmental stress by regulating the expression of transcription factors and target genes. Recent studies have shown that siRNA acts as a silencing signal in grafted plants, moving within the plant and mediating transcriptional [14] and post-transcriptional gene silencing [15]. Thus, it serves as a systemic signal that can epigenetically modify the recipient genome.

Transcriptome analysis in grafted plants can reveal the specific genes involved in regulating physiological responses induced by grafting, as well as elucidating differentially expressed genes (DEGs), the main metabolic pathways affecting plant growth and development, and the molecular mechanisms of plant trait changes [16,17,18]. Recent studies have shown that the leaf growth rate of grafted commercial potato varieties Hopehely and White Lady is directly proportional to the time of tuber formation and that differences in leaf metabolites are evident [19]. The grafting of early-maturing (Z5) and late-maturing (Z18) varieties of the Zhongshu 5 potato revealed the molecular mechanism of plant maturation and identified the related long-distance signaling molecules [20]. The present study grafted early- and late-maturing potato varieties homologously (self-grafting) and heterologously (onto each other). A transcriptome analysis of the grafted plants revealed DEGs and the main metabolic pathways that induce tuber formation in these early- and late-maturing varieties. *StYABBY1* was selected for functional verification from the key DEGs—overexpression resulted in smaller leaves and no potato tuber formation. This study provides new information for research into the genetic regulation of setting time in early- and late-maturing potato varieties.

## 2. Results

### 2.1. Heterologous Early-Maturing Scions Induce Potato Formation in Late-Maturing Rootstocks

**Figure 1:**
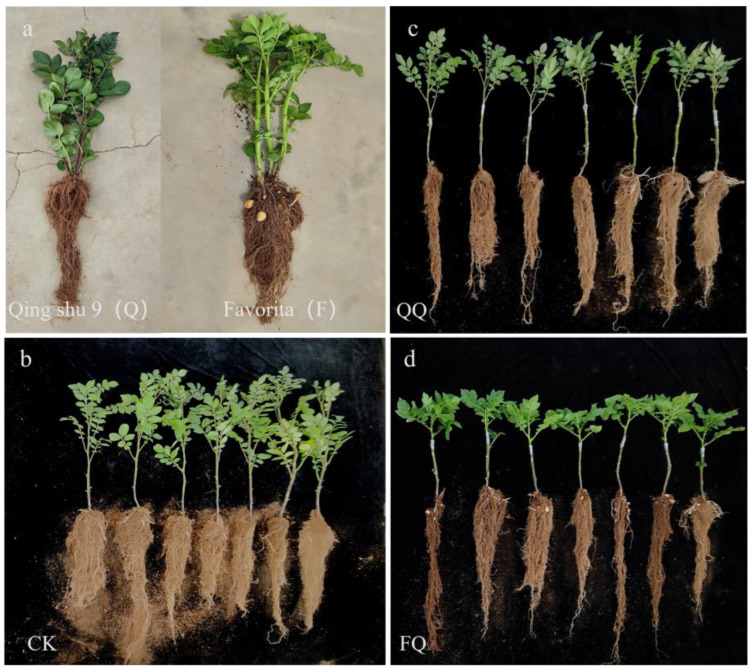
Phenotypes of reciprocally grafted potato plants after 18 d growth: (**a**) late-maturing variety Qingshu 9 (Q) in the budding stage and early-maturing variety Favorita (F) in the tuberization stage; (**b**) control variety Qingshu 9 (CK); (**c**) self-grafted Qingshu 9 (QQ); and (**d**) Favorita scion heterografted onto Qingshu 9 rootstock (FQ).

The early-maturing variety Favorita (F) in the potato-setting stage was selected as the scion and the late-maturing variety Qingshu 9 (Q) in the budding stage was selected as the rootstock for heterologous grafting (Figure 1a). This FQ combination (Figure 1d) exhibited earlier tuber formation than ungrafted, control plants (CK, all of which had stolons; Figure 1b) or QQ grafted plants (late-maturing scion grafted onto late-maturing rootstock; three out of seven of these plants had stolons; Figure 1c). Thus, heterologous early-maturing scions play an important role in inducing early potato formation in late-maturing rootstocks.

### 2.2. Transcriptome Sequencing

To investigate why early-maturing scions induce early tuber formation on late-maturing rootstocks, rootstocks were grafted at the early stage of potato formation. The BGISEQ sequencing platform was used for the transcriptome sequencing of 24 functional leaves and stolons of grafted plants. Samples produced an average of 46 M data, dropping to 44.30 M after filtering. The Q30 quality score was consistently above 91%. HISAT2 (v2.1.0) software was used to align clean reads to the reference genome GCF_000226075.1_SolTub_3.0 sequence. The average mapping rates of the FF, QQ, FQ, and QF leaves and stolons ranged from 72.2% to 82.0% (Table 1), showing that sequencing results were reliable and suitable for analysis.

### 2.3. Identification of DEGs

**Figure 2:**
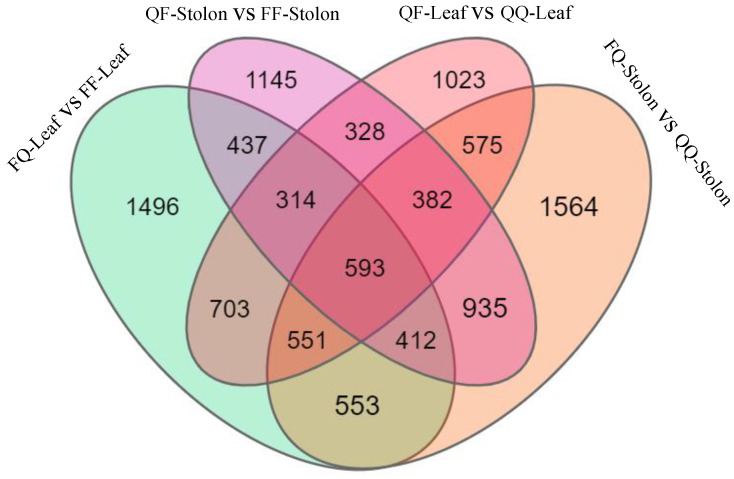
Venn diagram of differentially expressed genes.

The transcriptome sequencing of the 24 samples of leaves and stolons from heterologous (FQ, QF) and self-grafted (QQ, FF) plants was used to determine the DEGs involved in regulating tuber formation induced by grafting in early- and late-maturing varieties. The screening criteria were log2 (fold change) ≥ 0 and *p* < 0.5. The late-maturing variety Q exhibited 1496 genes in its rootstock leaves and 1145 genes in its scion creeping stem. The early-maturing variety F exhibited 1023 genes in its rootstock leaves and 1564 genes in its scion creeping stem. A total of 593 genes were upregulated in QF-leaf vs. QQ-leaf, FQ-stolon vs. QQ-stolon, FQ-leaf vs. FF-leaf, and QF-stolon vs. FF-stolon (Figure 2).

### 2.4. GO Enrichment of DEGs and KEGG Pathway Analysis

**Figure 3:**
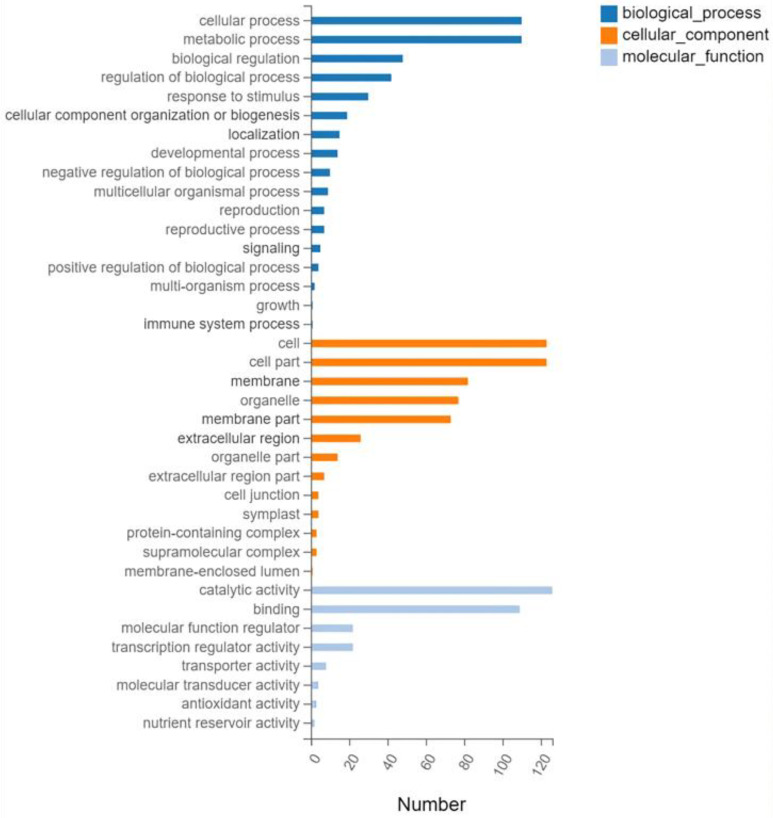
Gene ontology classifications.

**Figure 4:**
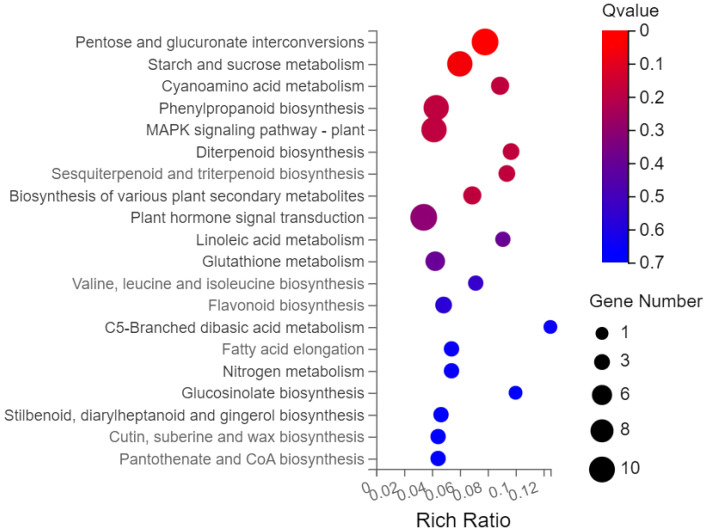
KEGG enrichment analysis bubble chart.

Gene ontology (GO) analysis was performed on the 593 screened DEGs (Figure 3). There were significant enrichments in 17 biological processes, 13 cellular components, and 8 molecular functions. Cellular processes and metabolic processes showed the highest degree of enrichment among the biological processes (110 genes each). The most enriched cellular component genes were related to cells (123), cell components (123), membranes (82), and organelles (77). The most enriched major molecular functions were catalytic activity (126 genes), binding (109), molecular function regulator (22), and transcription regulator activity (22). The Kyoto Encyclopedia of Genes and Genomes (KEGG) pathway analysis of the 593 DEGs showed enrichment predominantly in the interconversion of pentose and glucuronic acid, starch and sucrose metabolism, phenylpropanoid biosynthesis, plant hormone signal transduction, and plant MAPK signaling pathways, in addition to other pathways (Figure 4).

### 2.5. Identification of Transcription Factor Gene Families

Transcription factors (TFs) are a class of proteins with particular structures that play crucial roles in regulating plant growth and development. Changes in 38 TF genes were identified among the DEGs (classified in Supplementary Materials File S2 Excels). These included the bHLH TF phytochrome interacting factor 5 (*PIF5*), which is involved in photomorphogenesis, shade response, flowering time, and leaf senescence [21,22,23,24], and *bHLH93*, which regulates flowering time in *Arabidopsis* [25]. *CBF3* promotes leaf senescence and natural dormancy in fruit trees, improves plant resistance, and inhibits plant growth [26,27]. *ERF109* regulates jasmonic acid signaling and auxin biosynthesis during lateral root formation in *Arabidopsis* [26,27,28]. *TCP19* plays a redundant role in controlling leaf senescence [29], while the ectopic expression of *YABBY1* in rice plants leads to abnormal flowering period [30]. Based on transcriptome sequencing data, *StYABBY1* is highly expressed in potato leaves (Supplementary Materials File S1 Section 2). There are few reports on the functions of YABBY TFs in potato, so further exploration of the role of *YABBY1* is warranted.

### 2.6. Sequence Analysis and Subcellular Localization of Potato StYABBY1

**Figure 5:**
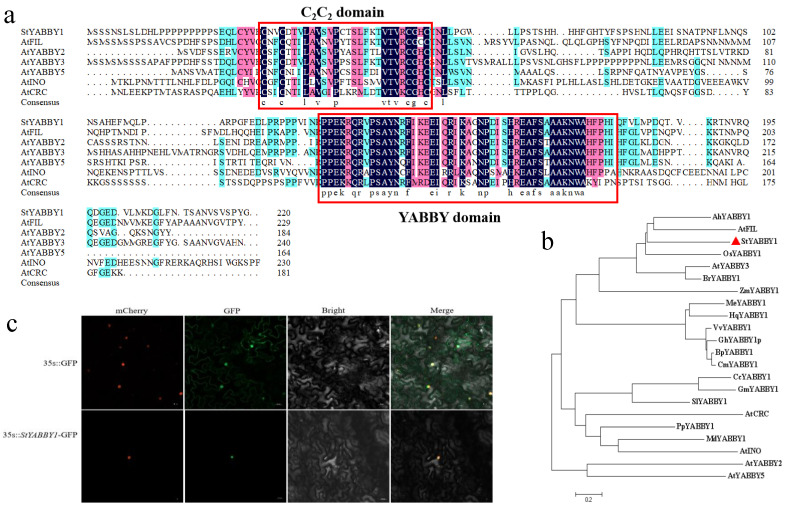
*StYABBY1* gene sequence analysis and subcellular localization. (**a**) Multiple sequence alignment of *StYABBY1* and *Arabidopsis StYABBY* proteins; (**b**) phylogenetic tree of *StYABBY1* and *YABBY1* proteins in other species; and (**c**) subcellular localization of *StYABBY1* protein in tobacco *(Nicotiana benthamiana)* epidermal cells.

*StYABBY1* is located on chromosome 1 of the potato genome. The open reading frame was 660 bp, encoding 219 amino acids, and its molecular weight was 24,454.66 kDa. Amino acid sequence comparison with six subfamilies of *Arabidopsis thaliana* showed that *StYABBY1* had a complete C2C2 zinc finger structure at the N terminus and a helix-loop-helix YABBY domain similar to the HMG structure at the C terminus (Figure 5a). Phylogenetic tree analysis showed that potato *StYABBY1* was located in the same evolutionary branch as the *Arabidopsis AtFIL*, peanut *AhYABBY1*, and rice *OsYABBY1* genes, and the evolutionary process was relatively conservative (Figure 5b). The tobacco transformation system was used to study the subcellular localization of *StYABBY1*. *StYABBY1*-GFP was prepared and introduced into tobacco leaves. Fluorescence microscopy showed that it co-localized with the nuclear marker mCherry in the nucleus, while control 35S::GFP was expressed in the entire epidermal cell nuclear membrane (Figure 5c).

### 2.7. StYABBY1 Overexpression Produces Smaller Leaves and Affects Tuber Formation

**Figure 6:**
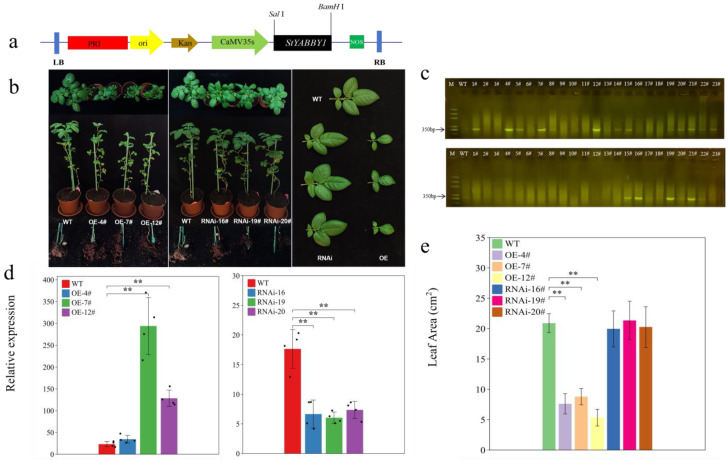
Effects of overexpressing *StYABBY1* on the leaves of potato plants. (**a**) Schematic diagram of pRI101-*StYABBY1* vector; (**b**) phenotypes of overexpression and RNAi transgenic plants at 60 d; (**c**) screening for kanamycin resistance by PCR electrophoresis; (**d**) relative expression of *StYABBY1* gene in transgenic plants; and (**e**) leaf area of transgenic plants.Note: # Number suffix, ** (*p* < 0.05).

Transgenic potato plants were produced using *StYABBY1* overexpression (OE) and RNA interference (RNAi) vectors to clarify the biological function of *StYABBY1* (Figure 6a). Resistance gene primers were designed and screened to identify transgenic plants by PCR (Figure 6c). To analyze the expression level of *StYABBY1* in various overexpression and interference plants, three overexpression lines were selected for RNA extraction and quantitative reverse transcription PCR with fluorescence detection. The expression of *StYABBY1* increased 1.49 to 12.74-fold in leaves of the overexpression lines compared to the wild type (WT), while expression in leaves of the interference plants fell by 2.39 to 2.92-fold (Figure 6d), demonstrating the success of both *StYABBY1* overexpression and RNA interference in these transgenic lines. Phenotypic analysis showed that the leaf area of *StYABBY1* overexpression plants was smaller than that of the wild type, while no tuber tubes were formed. By contrast, the RNAi line plants had tubers, but their leaf area was unaffected (Figure 6b,e). It can, therefore, be inferred that the *StYABBY1* gene plays an important role in potato leaf development and tuber formation.

## 3. Discussion

Grafting experiments have been previously used to identify mobile signals that induce tuberization [31]. This study conducted grafting experiments to identify the key DEGs responsible for tuber induction in early- and late-maturing potato varieties and validated the biological functions of candidate genes.

Heterografting and reciprocal grafting were performed on the tetraploid early-maturing potato variety Favorita and the late-maturing variety Qingshu 9 under normal growth conditions. Heterografting revealed the effect of early-maturing scions on the tuberization of late-maturing rootstocks: the rootstocks were induced to tuberize earlier. The phenotypic changes induced by grafting resulted from the interaction between scion and rootstock. This is consistent with previous reports that tobacco flowering scions grafted onto potatoes promoted tuberization in the rootstock, suggesting that flowering and tuberization signals are similar [32]. The main drivers of potato tuber formation are the homologs of the flowering gene *FT*, *StSP6A*, and *StCO* [33]. Under appropriate conditions, *StSP6A* is activated and expressed in the leaves, and then the protein is transported to the tip of the stolons and induces tuber formation [11]. This study provides new insight into differences in the timing of tuberization between early- and late-maturing varieties by investigating the interaction between scion and rootstock.

Most early-maturing potato varieties exhibit small above-ground plants, large leaves, early flowering, and early tuber formation compared to late-maturing varieties. Genetic studies of potato maturation indicate that these traits are controlled by multiple genes. The main site of action—*StCDF1*, located on chromosome 5—contains 12 unique alleles. *StCDF1.1* is the allele for late maturation, while *StCDF1.2* and *StCDF1.3* are alleles for early maturation [34,35]. Potato maturity is associated with initial tuberization time. Plants that tuberize early usually exhibit early maturation. Only a few signals, such as *StSP3D* and *StSP6A*, have been related to potato tuberization [36,37], but whether they positively or negatively affect maturation is currently unclear. This study screened 593 upregulated genes and found that *PIF5*, *bHLH93*, *CBF3*, *ERF109*, *TCP19*, and *YABBY1* may be key genes in the induction of tuber formation in early- and late-maturing potatoes. The late-maturing *StCDF1.1* and early-maturing *StCDF1.2* and *StCDF1.3* genes were not among these 593 genes. However, the key differential gene *CBF3* and the long-distance mobile signal molecules linked to potato maturation (*StCBF1* and *StCBF2*) belong to the same family [20], so the previously unreported key differential gene *YABBY1* was selected for further functional and molecular mechanism analysis.

The YABBY transcription factor family has various biological roles in higher plant growth and development. Its members regulate key functions in the development of above-ground lateral organs such as leaves and flowers [38,39], secondary metabolite formation [40,41,42], and the maintenance of meristematic tissue activity [43,44]. In recent years, the YABBY family has been identified and investigated in an increasing number of species, and its functions have been determined. The overexpression of YABBY-homologous genes led to abnormal development of floral organs and leaves in *Arabidopsis* [43], rice [45], and maize (*Zea mays*) [44]. The sequence of the *StYABBY1* gene was cloned from potatoes in the present study. The gene produced an amino acid sequence containing a complete C2C2 zinc finger and YABBY domains. It belongs to the filamentous flower (*FIL*) subfamily, which is similar to proteins in *Arabidopsis*, tomato, and other dicotyledonous species [46,47]. Phylogenetic analysis showed that *StYABBY1* is closely homologous in *Arabidopsis*, peanut, and rice. This high evolutionary conservation suggests that the gene might have similar functions in these plants. *OsYAB1* ectopic expression in rice resulted in normal vegetative growth and abnormal flowering periods. The number of stamens and carpels in the spikelets increased compared to wild-type plants [30]. The ectopic expression of *TaYAB1*, *BraYAB1-702*, and *GmFILa* in *Arabidopsis* led to leaf curling and delayed flowering [48,49,50]. This study showed that *StYABBY1* transcription factor is a repressor of leaf growth and tuber formation in potato. These findings confirm that *StYABBY1* regulates leaf size and plays important biological roles in potatoes, but its pathways require further study.

## 4. Materials and Methods

### 4.1. Plant Materials, Growth Conditions, and Grafting

Late-maturing potato variety Qingshu 9 (Q; growth period 120 d) and early-maturing variety Favorita (F; growth period 60 d) were utilized in heterografting experiments conducted in greenhouses of the Biotechnology Research Institute of the Qinghai Academy of Agriculture and Forestry Sciences (Xining, China). Healthy, undamaged potato tubers were planted on 27 April 2023, in pots of 58 cm height and 40 cm diameter filled with a mixture of nutrient soil and substrate. One hundred pots were prepared for each variety and seedlings were fixed on 6 July 2023, with one plant per pot. Ungrafted Q was used as a control, with the treatments being self-grafted QQ and heterografted FQ (QQ: scion and rootstock from different Qingshu 9 plants; FQ: ungrafted Favorita with Qingshu 9 as the rootstock). Each combination was replicated in 24 randomly arranged pots. Grafting was performed using the cleft method on 15 July 2023: a 3–4 cm vertical incision was made in the rootstock, and the scion was inserted into the incision, fixed immediately with breathable sealing film, and then covered with a self-sealing bag to conserve moisture. The bag was removed after 7 d and the tuberization status was recorded after 18 d of growth.

A reciprocal grafting experiment was conducted in an artificial climate chamber at the Biotechnology Research Institute of the Chinese Academy of Tropical Agricultural Sciences. Healthy, undamaged potato tubers were planted on 27 March 2022, in pots of 18 cm height and 23 cm diameter filled with a mixture of nutrient soil and substrate. The light intensity was 5000 Lx, temperature 23 °C/20 °C, photoperiod 12 h/day, and humidity 45%. After emergence, one plant with 7–10 leaves was cultivated in each pot. Grafting was performed when the plant reached 40–50 cm height. Self-grafted FF and QQ were used as controls (FF: scion and rootstock from different Favorita plants; QQ: scion and rootstock from different Qingshu 9 plants), with the treatments being heterografted FQ and QF (FQ: Favorita as scion, Qingshu 9 as rootstock; QF: Qingshu 9 as scion, Favorita as rootstock). Each of the four grafted combinations was replicated 12 times. Grafting was performed on 10 May 2022, using the cleft method and surviving plants were cultivated until death.

### 4.2. Sample Collection and Preparation

Functional leaves and stolons of self-grafted FF and QQ and heterografted FQ and QF plants were sampled from the second to third leaves down from the top (three biological replicates of each). Soil and dust were washed off with distilled water before samples were blotted dry with filter paper, cut into small pieces (approximately 50–100 mg), transferred to 5 mL capped centrifuge tubes, immediately frozen with liquid nitrogen, and stored at −80 °C before sending on dry ice to BGI Genomics for transcriptome sequencing.

### 4.3. RNAseq Sample and Library Preparation

Grafted plant tissue samples of FF-leaf, FF-stolon, QQ-leaf, QQ-stolon, FQ-leaf, FQ-stolon, QF-leaf, and QF-stolon were collected. RNA extraction and transcriptome sequencing of three biological replicates were conducted by BGI Genomics using the BGISEQ sequencing platform.

### 4.4. Sequence Data Analysis

Raw sequencing data were filtered using SOAPnuke (v.1.5.6) [51] to remove (i) reads containing adapters (adapter contamination), (ii) reads with an unknown base N content greater than 5%, and (iii) low-quality reads (quality value below 15 accounting for more than 20% of the total bases in the read). The analysis and plotting of the clean data were performed using the Dr.Tom multi-omics data mining system (https://biosys.bgi.com). Differential gene analysis was conducted using HISAT2 (v.2.1.0) [52] to align the clean data to the reference genome, and Bowtie2 (v.2.3.4.3) [53] to align the clean data to the reference gene set (provided by the Dr.Tom system). Gene expression quantification used RSEM (v.1.3.1) [54] and heatmaps were drawn using pheatmap (v.1.0.8) [55]. Differential gene detection was performed using DESeq2 (v.1.4.5) [56], applying a Q value threshold of ≤0.05. KEGG and GO enrichment analyses based on hypergeometric distributions were used to explore the functional differential genes associated with phenotypic changes (Q value threshold ≤ 0.05 [57]). Genes meeting this condition were defined as significantly enriched in candidate genes.

### 4.5. Cloning, Multiple Sequence Alignment, and Phylogenetic Relationships of StYABBY1

Total RNA was extracted from Désirée potato leaves using the RNAprep Pure Plant Total RNA Kit (Tiangen, Beijing, China; www.transgen.com.cn). cDNA was synthesized using the MonScript RT Super Mix with dsDNase protocol (Monad, Wuhan, China; www.monadbiotech.com). The *StYABBY1* sequence was downloaded from the Phytozome database (https://phytozome-next.jgi.doe.gov/). The full-length coding sequence (CDS) of *StYABBY1* was amplified using gene-specific PCR primers (Supplementary Material File S1 Sections 1–7). Homologous amino acid sequences of *StYABBY1* from various species were downloaded from the National Center for Biotechnology Information (NCBI) database. Multiple sequence alignment was performed in DNAMAN 7.0 and a phylogenetic tree was constructed using the neighbor-joining method in mega 6.0.

### 4.6. Subcellular Localization of StYABBY1

The *Agrobacterium* strain GV3101 (pSoup-p19) carrying the pCAMBIA1300-*StYABBY1*-GFP fusion plasmid and the pCAMBIA1300-GFP plasmid were transiently transformed into tobacco *(Nicotiana benthamiana)* epidermal cells and dark-cultivated for 24 h before transferring to a light incubator for 48–72 h. The green fluorescent protein (GFP) signal was observed using a laser confocal microscope (Zeiss LSM800).

### 4.7. Potato Genetic Transformation

The CDS of *StYABBY1* was cloned from Désirée potato leaves, and Sal1 and BamH1 were chosen as restriction enzyme cutting sites. The pRI101-*StYABBY1* vector was constructed and activated, and correctly sequenced clones were extracted and transferred into *Agrobacterium* GV3101. The complete vector was transformed into potatoes using the *Agrobacterium*-mediated transformation method as described previously [58].

### 4.8. Phenotypic Identification of Transgenic Plants

Successful transgenic plants were propagated on standard MS medium supplemented with Timentin (500 μL/100 mL). After 25 d of growth, lids were removed in an LB-QHS artificial climate chamber (temperature 22 °C/16 °C, light intensity 5000 Lx, photoperiod 12 h/12 h, humidity 45%) and, after acclimatizing for 5 d, plants were transplanted into pots containing nutrient soil. Tuberization was recorded after 60 d, the length and width of functional leaves were measured, and leaf area was estimated using a non-destructive estimation model [59].

### 4.9. Real-Time Quantitative PCR (qRT-PCR) Analysis

Total RNA extraction and cDNA synthesis were performed using the Tiangen extraction kit and the Monad MonScript RT Super Mix with dsDNase kit. Primers were designed using Primer Premier 6.0 software (Supplementary Material File S1 Section S8). The qRT-PCR program was set according to the required annealing temperature and cycle number. The internal reference gene was β-actin. Relative expression levels were determined using the (Ct) 2^−ΔΔCt^ method, with four biological replicates.

### 4.10. Data Analysis

SPSS 17.0 (IBM) was used for data analysis. Comparisons between wild-type and transgenic lines were made using Student’s *t*-test, with significance thresholds at *p* < 0.05 and *p* < 0.01. Graphs were created using WPS 2019 and GraphPad Prism 9.5 software.

## 5. Conclusions

This study identified 593 DEGs related to tuberization induction in early- and late-maturing potato varieties, partially corroborating previous reports on the functioning of genes related to tuber formation. Specifically, *PIF5*, *bHLH93*, *CBF3*, *ERF109*, *TCP19*, and *YABBY1* were identified as potential key genes inducing tuberization. The StYABBY1 gene was cloned from potatoes and encoded a typical C2C2 zinc finger domain and YABBY domain TF of the FIL subfamily. The biological functions of *StYABBY1* in regulating leaf size and tuber development were studied, shedding further light on the regulatory mechanisms of tuber formation and suggesting that gene engineering is a possible route to managing potato growth stages and tuberization.

## Figures and Tables

**Table 1:** Transcriptome sequencing quality statistics.

Sample	Raw Reads	Clean Reads	Q30 (%)	Total Mapped (%)	Uniquely Mapped (%)
FF-leaf	45.57	44.65	91.2	72.2	69.9
FF-stolon	45.57	44.69	91.1	74.2	71.7
QQ-leaf	45.57	44.59	93.1	79.4	76.9
QQ-stolon	45.57	44.44	94.2	81.9	79.4
FQ-leaf	45.57	44.34	94.1	82.0	79.3
FQ-stolon	45.57	44.33	94.1	81.2	78.6
QF-leaf	45.57	44.37	94.2	81.0	78.4
QF-stolon	45.57	44.50	93.8	81.3	78.7

## Data Availability

All data supporting the conclusions of this article are provided within the article and its Supplementary Information. The transcriptome data used in the current study are available in the National Center for Biotechnology Information (NCBI) Sequence Read Archive (SRA) database (https://www.ncbi.nlm.nih.gov/sra) (accession number: PRJNA1102724) (accessed on 25 June 2024).

## Supplementary Materials

The following supporting information can be downloaded at: https://www.mdpi.com/article/10.3390/plants13131879/s1, Supplementary Materials File S1: 1. Primers used in this study; 2. Cloning sequence of *StYABBY1* gene; 3. iRNA target sequence; 4. Multiple sequence alignment of original sequences; 5 Primitive sequence of phylogenetic tree; 6. Original qPCR data of genetically modified plants; 7. Statistical data on leaf area of genetically modified plants; 8. Classification and functional annotation of transcription factors; Supplementary Materials File S2: S1. DEGs-593; S2. DEGs-Gen expression.
