# Supplementary material for: Transcriptomics Identifies Differentially Expressed Genes Inducing Tuber Formation in Early- and Late-Maturing Potatoes
Source: Plants (Basel). 2024 Jul 8;13(13):1879. doi: 10.3390/plants13131879 (PMC11243988; doi:10.3390/plants13131879)
Supplement: Supplementary file 1 [file plants-13-01879-s001.zip › plants-3021416-Supplementary Materials-S1.pdf]

Supplementary Materials

1、Primers used in this study

	Primer sequence	
**Primer Name**	**Sequence(5`-3`)**	**Use for**
StYABBY1OE-F/SalI	ACGCGTCGACATGTCCTCTTCAAATAGCTTGTC	PRI101
StYABBY1OE-R/BamHI	CGCGGATCCTCAGTAAGGAGACACACTAAC	
StYABBY1GFP-F/SalI	ACGCGTCGACATGTCCTCTTCAAATAGCTTGTC	pCAMBIA1300
StYABBY1GFP-R/BamHI	CGCGGATCCGTAAGGAGACACACTAAC	
StYABBY1-qF	CCTCCTCCGCCTCCACCTC	qRT-PCR
StYABBY1-qR	TGTCCACATCGAACCGTCACAG	
Kana-F	ACTCCATCGACATATCGG	Identify transgenic plants
Kana-R	TAGCTCGACATACTGTTCT	

2、Cloning sequence of StYABBY1 gene

>S.tuberosumv4.03|PGSC0003DMG400004741Sequencing results：

ATGTCCTCTTCAAATAGCTTGTCATTGGACCACCTTCCTCCTCCTCCGCCTCCACCTCCTTCCGAGCAGCTCTGCTATGTCCATTGCAACGTTTGTGACACCGTCCTCGCGGTAAGTGTACCGTGCACAAGTTTGT

TCAAAACTGTGACGGTTCGATGTGGACACTGCACTAATCTTTTGCCTGGATGGCTTTTGCCTTCCACTAGTCATCATCATCATTTTGGTCACACTTACTTCTCTCCTTCCCACAATCTTCTGGAAGAAATAAGCAATG

CAACCCCAAATTTCTTGATGAATCAGAGTAACTCAGCTCATGAATTTATGCAGCTGCCTGCTCGACCTGGATTTGAGGATCTTCCTAGACCGCCACCCGTTATTAACAGACCTCCTGAGAAGAGACAGCGAGTCCCT

TCTGCTTACAACCGATTCATCAAGGAAGAAATCCAACGCATAAAAGCAGGGAATCCTGATATTAGCCACAGAGAAGCCTTCAGCGCCGCTGCAAAAAATTGGGCCCACTTTCCACACATTCAATTTGTTCTCATGC

CTGATCAGACTGTTAAGAGGACTAATGTGCGTCAGCAGGATGGAGAAGATGTTCTTATGAAAGATGGTTTGTTTAATACATCAGCCAATGTTAGTGTGTCTCCTTACGGA

3、iRNA target sequence

No.	Sequence	Start	GC%	Scores	ΔE/Thermodynamic	SNPs	Off-target	Pos-Motifs
1	AAGAGGACTAATGTGCGTCAG	567	47.62	19.5	2.90/-36.40	NA	0/	0
2	AACTGTGACGGTTCGATGTGG	146	52.38	14.96	0.33/-37.60	NA	0/	0
3	AATTTGGTCTCATGCCTGATC	538	42.86	12.15	0.23/-35.30	NA	0/	0

4、Multiple sequence alignment of original sequences

Amino acid multiple sequence analysis	
>StYABBY1	MSSSNSLSLDHLPPPPPPPPPSEQLCYVHCNVCDTVLAVSVPCTSLFKTVTVRCGHCTNLLPGWLLPSTSHHHHFGHTYFSPSHNLLEEISNATPNFLMNQSNSAHEFMQLPARPGFE DLPRPPPVINRPPEKRQRVPSAYNRFIKEEIQRIKAGNPDISHREAFSAAAKNWAHFPHIQFVLMPDQTVKRTNVRQQDGEDVLMKDGLFNTSANVSVSPYG
>AtFIL	MSMSSMSSPSSAVCSPDHFSPSDHLCYVQCNFCQTILAVNVPYTSLFKTVTVRCGCCTNLLSVNMRSYVLPASNQLQLQLGPHSYFNPQDILEELRDAPSNMNMMMMNQHPTMN DIPSFMDLHQQHEIPKAPPVNRPPEKRQRVPSAYNRFIKEEIQRIKAGNPDISHREAFSAAAKNWAHFPHIHFGLVPDNQPVKKTNMPQQEGEDNMVMKEGFYAPAAANVGVTPY
>AtYABBY2	MSVDFSSERVCYVHCSFCTTILAVSVPYASLFTLVTVRCGHCTNLLSLNIGVSLHQTSAPPIHQDLQPHRQHTTSLVTRKDCASSSRSTNNLSENIDREAPRMPPIRPPEKRQRVPSAY NRFIKEEIQRIKACNPEISHREAFSTAAKNWAHFPHIHFGLKLDGNKKGKQLDQSVAGQKSNGYY
>AtYABBY3	MSSMSMSSSSAPAFPPDHFSSTDQLCYVHCSFCDTVLAVSVPPSSLFKTVTVRCGHCSNLLSVTVSMRALLLPSVSNLGHSFLPPPPPPPPPNLLEEMRSGGQNINMNMMMSHHAS AHHPNEHLVMATRNGRSVDHLQEMPRPPPANRPPEKRQRVPSAYNRFIKEEIQRIKAGNPDISHREAFSAAAKNWAHFPHIHFGLMADHPPTKKANVRQQEGEDGMMGREGFYG SAANVGVAHN
>AtYABBY5	MANSVMATEQLCYIPCNFCNIILAVNVPCSSLFDIVTVRCGHCTNLWSVNMAAALQSLSRPNFQATNYAVPEYGSSSRSHTKIPSRISTRTITEQRIVNRPPEKRQRVPSAYNQFIKEEI QRIKANNPDISHREAFSTAAKNWAHFPHIHFGLMLESNKQAKIA
>AtINO	MTKLPNMTTTLNHLFDLPGQICHVQCGFCTTILLVSVPFTSLSMVVTVRCGHCTSLLSVNLMKASFIPLHLLASLSHLDETGKEEVAATDGVEEEAWKVNQEKENSPTTLVSSSDNE DEDVSRVYQVVNKPPEKRQRAPSAYNCFIKEEIRRLKAQNPSMAHKEAFSLAAKNWAHFPPAHNKRAASDQCFCEEDNNAILPCNVFEDHEESNNGFRERKAQRHSIWGKSPFE
>AtCRC	MNLEEKPTMTASRASPQAEHLYYVRCSICNTILAVGIPLKRMLDTVTVKCGHCGNLSFLTTTPPLQGHVSLTLQMQSFGGSDYKKGSSSSSSSSTSSDQPPSPSPPFVVKPPEKKQRL PSAYNRFMRDEIQRIKSANPEIPHREAFSAAAKNWAKYIPNSPTSITSGGHNMIHGLGFGEKK

5、Primitive sequence of phylogenetic tree

>StYA BBY1	MSSSNSLSLDHLPPPPPPPPPSEQLCYVHCNVCDTVLAVSVPCTSLFKTVTVRCGHCTNLLPGWLLPSTSHHHHFGHTYFSPSHNLLEEISNATPNFLMNQSNSAHEFMQLPARPGFEDLPRPPPVINRPPEKRQR VPSAYNRFIKEEIQRIKAGNPDISHREAFSAAAKNWAHFPHIQFVLMPDQTVKRTNVRQQDGEDVLMKDGLFNTSANVSVSPYG
>BpYA BBY1	MSSSSTLSLDHLPPSEQLCYVHCNICDTVLAVSVPCTSLFKTVTVRCGHCTNLLPVNMRGLLLPSANQFHLGHSFFSSSHNLLEEIPNPTPNFLINQTNASEFTMPARGGVDELPRPPVINRPPEKRQRVPSAYNR FIKDEIQRIKSVNPDISHREAFSAAAKNWAHFPHIHFGLMPDQTVKKTNVRQQEGEDVLMKDGYFASANVGVSPY
>SlYA BBY1	MSSSNSLSLDHLPPPPPSEQLCYVHCNVCDTVLAVSVPCTSLFKTVTVRCGHCTNLLPGWLLPSTNHHHHHFGHTYFSPSHNLLDEISNATPNFLMNQSNSAHEFVQLPARPGFDDLPRPPPVVNRPPEKRQRV PSAYNRFIKEEIQRIKAGNPDISHREAFSAAAKNWAHFPHIQFGLMPDQTVKRTNVRQQDGEDVLTKDGLFNTSANVSVSPY
>VvYA BBY1	MSSSSTLSLDHLPPSEQLCYVHCNICDTVLAVSVPCTSLFKTVTVRCGHCTNLLPVNLRGLLLPSANQLHLGHAFFSPSHNLLEEIPNPSPNFLINQTTANDFSVSARGGADELPRPPVINRPPEKRQRVPSAYNR FIKDEIQRIKAGNPDITHREAFSAAAKNWAHFPHIHFGLMPDQTMKKTNVRQQEGEDVLMKDGFFASANVGVSPY
>MeY ABBY 1	MSSSSTLSLDHLPPSEQLCYVQCNICNTVLAVSVPCTSLFKTVTVRCGHCTNLLPVNMRGLILPSANQFHLGHSFYSPSHNLLDEIPNPSPNFLINQTNANDFSVPARGVTNDELPRPPVINRPPEKRQRVPSAYN RFIKDEIQRIKAGNPDISHREAFSAAAKNWAHFPHIHFGLMPDQTVKKTNVRQQEGEDVLMKDGFFASANVGVSPY
>GhYA BBY1p	MSSSSTLSLDHLPPSEQLCYVHCNICDTVLAVSVPCTSLFKTVTVRCGHCTNLLPVNMRGLLMPSANQFHLPHNFFTPSHNLLEEISNPSPNILLNQGNTSDITLPTRGVADELPRPPVINRPPEKRQRVPSAYNR FIKDEIQRIKAGNPDITHREAFSAAAKNWAHFPHIHFGLMPDQPAKRTNVRQQEGEDVLMKDGFFASANVGVTPY
>CmY ABBY 1	MSSSSTLSLDHLPPSEQLCYVHCNICDTVLAVSVPCTSLFKTVTVRCGHCTNLLPVNMRGLLLPSANQFHLGHSFFSPSHNLLEEIPNPSPNFLINQNNGSDFTMPARGGVGELPRPPAINRPPEKRQRVPSAYNR FIKDEIQRIKSVNPDISHREAFSAAAKNWAHFPHIHFGLMPDQTVKKTNVRQEGEDVLMKDGFFASADVGVSPY
>HqYA BBY1	MSSSSTLSLDHFPPSEQLCYVHCTICDTVLAVSVPCSSLFKTVTVRCGHCTNLLPVNMRGLLLPSANQLHIGHSFFSPSHNFMEDIPNPTPNFVLNQTNPNDFSLPARGGLDHGQLPRPPVINRPPEKRQRVPSAY NRFIKDEIQRIKAGNPDISHREAFSAAAKNWAHFPHIHFGLMPDQPVKKANVRQQEGEDVLMKDGFFASANVGVSPY
>AhYA BBY1	MSSSSSSSSSSTTLSLDHIPPSEQLCYVHCNICDTVLAVSVPCTSLFKTVTVRCGHCTNLLPVNMRGLLLPSPNQFHFGHSFFSPTHNLLEEIPNPSPNFFMNQTNTPNEFSMPTRTVVDELPRPPIINRPPEKRQRV PSAYNRFIKDEIQRIKSVNPDITHREAFSAAAKNWAHFPHIHFGLMPDQTVKKTNVRQQEGEEVLMKDAGFYASANVGVSPY
>CcYA	MSSSSTTLSLDHLPPSEQLCYVHCNICDTVLAVSVPCTSLFKTVTVRCGHCTNLLPVNMRGLLLPSPNQFHLGHSLFSPSHNLLEEMPNPSPNFLINQTNSSNDCSMPARTAADELPRPPVINRPPEKRQRVPSAY

BBY1	NRFIKDEIQRIKSVNPDITHREAFSAAAKNWAHFPHIHFGLMPDQTVKKTNVRQQEGEDVLMKDGFFASANVRVSPY
>PpYABBY1	MSSSSSASTLSLDHLPPSEQLCYVHCNICDTVLAVSVPCTSLFKTVTVRCGHCTNLLPVNMRGLLLPSPNQFHHLGHSFFSSPNNSHNLLEEIPNPAPNFLMNQTSVNDFAVRPRGGADELPRPPAITRPPEKRQRVPSAYNRFIKDEIQRIKSVNPDISHREAFSAAAKNWAHFPHIHFGLMPDQTVKKTNVRQQEGEDVLMKDGFFASANNVRVSPY
>MdYABBY1	MSSSSASTLSLDHLPPSEQLCYVHCNICDTVLAVSVPCTSLFKTVTVRCGHCTNLLPVNMRGLLLPSPNQFHHLGHSFFSSPSNSHNLLEEIPNVPTPNFLMNQTSVNDFAARPRGGPDELLPRPPVINRPPEKRQRVPSAYNRFIKDEIQRIKSVNPDISHREAFSAAAKNWAHFPHIHFGLMPDQTVKKTTVRQQEGEDVVMKDGFFSSANNVRVLPY
>AtFIL	MSMSSMSSPSSAVCSPDHFSPSDHLCYVQCNFCQTILAVNVPYTSLFKTVTVRCGCCTNLLSVNMRSYVLPASNQLQLQLGPHSYFNPQDILEELRDAPSNMNMMMMNQHPTMNDIPSFMDLHQQHEIPKAPPVNRPPEKRQRVPSAYNRFIKEEIQRIKAGNPDISHREAFSAAAKNWAHFPHIHFGLVPDNQPVKKTNMPQQEGEDNMVMKEGFYAPAAANVGVTPY
>AtYABBY2	MSVDFSSERVCYVHCSFCTTILAVSVPYASLFTLVTVRCGHCTNLLSLNIGVSLHQTSAPPIHQDLQPHRQHTTSLVTRKDCASSSRSTNNLSENIDREAPRMPPIRPPEKRQRVPSAYNRFIKEEIQRIKACNPEISHREAFSTAAKNWAHFPHIHFGLKLDGNKKGKQLDQSVAGQKSNGYY
>AtYABBY3	MSSMSMSSSSAPAFPPDHFSSTDQLCYVHCSFCDTVLAVSVPPSSLFKTVTVRCGHCSNLLSVTVSMRALLLPSVSNLGHSFLPPPPPPPPNLLEEMRSGGQNINMNMMMSHHASAHHPNEHLVMATRNGRSVDHLQEMPRPPPANRPPEKRQRVPSAYNRFIKEEIQRIKAGNPDISHREAFSAAAKNWAHFPHIHFGLMADHPPTKKANVRQQEGEDGMMGREGFYGSAANVGVAHN
>AtYABBY5	MANSVMATEQLCYIPCNFCNIILAVNVPCSSLFDIVTVRCGHCTNLWSVNMAAALQSLSRPNFQATNYAVPEYGSSSRSHTKIPSRISTRTITEQRIVNRPPEKRQRVPSAYNQFIKEEIQRIKANNPDISHREAFSTAAKNWAHFPHIHFGLMLESNKQAKIA
>AtINO	MTKLPNMTTTLNHLFDLPGQICHVQCGFCTTILLVSVPFTSLSMVVTVRCGHCTSLLSVNLMKASFIPLHLLASLSHLDETGKEEVAATDGVEEEAWKVNQEKENSPTTLVSSSDNEDEDVSRVYQVVNKPPEKRQRAPSAYNCFIKEEIRRLKAQNPSMAHKEAFSLAAKNWAHFPPAHNKRAASDQCFCEEDNNAILPCNVFEDHEESNNGFRERKAQRHSIWGKSPFE
>AtCRC	MNLEEKPTMTASRASPQAEHLYYVRCSICNTILAVGIPLKRMLDTVTVKCGHCGNLSFLTTTPPLQGHVSLTLQMQSFGGSDYKKGSSSSSSSSTSSDQPPSPSPPFVVKPPEKKQRLPSAYNRFMRDEIQRIKSANPEIPHREAFSAAAKNWAKYIPNSPTSITSGGHNMIHGLGFGEKK
>OsYABBY1	MSSSSSSSAVFPLDHLAAPSPTEQLCYVHCNCCDTILAVGVPCSSLFKTVTVRCGHCANLLSVNLRGLLLPAPAPAPANQLHFGPSLLSPTSPHGLLDEVAFQTPSLLMEQAASASLSSITGRSSSSCASNAPAMQMPPAKPVQQEPELPKNAPASANRPPEKRQRVPSAYNRFIKDEIQRIKAGNPDISHREAFSAAAKNWAHFPHIHFGLMPDQGFKKTFKPQDGSEDILLKDSLYAAAAAAAAAAANMGVTPF
>ZmYABBY1	MMSSSSSSSAACCFPLDHLAPSPTEQLCYVHCNCCDTILAVGVPCSSLFKTVTVRCGHCANLLSVNLRGLLLPPAAPAPPNHLNFAHSLLSPTSPHGLLDELALQQAPSFLMEQASANLSSTMTGRSSNSSCASNLPPPAPMPAAQPVQQEAELPKTAPSVNRPPEKRQRVPSAYNRFIKDEIQRIKAGNPDITHREAFSAAAKNWAHFPHIHFGLMPDQGLKKTFKTQDGAEDMLLKDDLYAAAAAAAAANMGITPF
>GmYABBY1	MSSSSSTLSLDHLPPSEQLCYVHCNICDTVLAVSVPCTSLFKTVTVRCGHCTNLLPVNMRGLLMPSPTQFHLGHSFFSPSHNLLEEIPNPTPNFLMNQTNFSASHEFSMPARTAADELPRPPITNRPPEKRQRVPSAYNRFIKDEIQRIKSVNPDITHREAFSAAAKNWAHFPHIHFGLMPDQTVKKTNVCQQDGEEVLMKDGFYASANVGVSPY

>BrYA<br>BBY1	MSSMSMSSSSAPAYPPDHISSSDQLCYVHCSFCDTVLAVSVPPSSLFKTVTVRCGHCSNLLSVTVNMRALLLPSVSNIGHSFLPSPPPPPPPNLLEEMRNGGQNINMNMMMSHHAAAHHSNESFVMATRVRSV<br>DLQEMPRPPPANRPAPEKRQRVPSAYNRFIKEEIQRIKAGNPDISHREAFSAAAKNWAHFPHIHFGLMPDHPPTKKANVRQQEGEEVMMGREGFYGSAANVGVTHN

6、Original qPCR data of genetically modified plants

Sample Name	Relative expression level 1	Relative expression level 2	Relative expression level 3	Relative expression level 4
CK	16.60857268	19.34456242	29.73017788	26.60925456
OE-4	32.85142283	26.60925456	47.30288234	30.9926925
OE-7	313.8336392	215.8456473	370.6352248	275.3211443
OE-12	125.748073	118.0992661	114.0763716	155.8329159
CK	19.21093977	12.94081155	20.30630991	18.17465647
RNAi-16	8.657936147	5.111121893	4.179550863	8.689124839
RNAi-19	5.519670193	6.290391385	5.107296751	7.262337371
RNAi-20	5.336743126	7.378249677	8.641747462	8.106987759

7、Statistical data on leaf area of genetically modified plants

Sample Name	Leaf length	Leaf width	Leaf area	Leaf length	Leaf width	Leaf area	Leaf length	Leaf width	Leaf area	Leaf length	Leaf width	Leaf area	Leaf length	Leaf width	Leaf area
CK	5.4	4	20.088	5.8	4.3	23.1942	5.7	3.9	20.6739	5.5	4.2	21.483	5.7	3.6	19.0836
OE-4	3.3	2.5	7.6725	4	2.1	7.812	2.9	1.8	4.8546	3.8	2.5	8.835	3.7	2.6	8.9466
OE-7	3.5	2.7	8.7885	4.1	2.8	10.6764	3.8	2	7.068	4	2.5	9.3	3.5	2.5	8.1375
OE-12	3.5	2.2	7.161	3.5	2	6.51	2.8	1.6	4.1664	2.7	1.7	4.2687	2.6	1.9	4.5942
RNAi-16	5.6	4.2	21.8736	5.9	4	21.948	5.4	3.4	17.0748	5.9	4.1	22.4967	5.7	3.1	16.4331
RNAi-19	5.8	4.4	23.7336	5.4	4	20.088	5.7	4.1	21.7341	6	4.4	24.552	5.1	3.5	16.6005
RNAi-20	5.2	3.6	17.4096	5.7	4.3	22.7943	5.8	4.3	23.1942	5.5	4.3	21.9945	5.2	3.3	15.9588

8. Classification and functional annotation of transcription factors.

TF classification	Gene ID	Chr	Gene name	Gene molecular function
bHLH	LOC102588413	Chr04	transcription factor PIF5-like	protein dimerization activity
	LOC102592363	Chr04	transcription factor HEC2-like	DNA-binding transcription factor activity
	LOC102592498	Chr02	transcription factor bHLH18-like	protein dimerization activity
	LOC102592871	Chr07	bHLH66	DNA-binding transcription factor activity, RNA polymerase II
	LOC102594599	Chr04	bHLH96-like	DNA-binding transcription factor activity, RNA polymerase II
	LOC102597520	Chr04	transcription factor bHLH93-like	sequence-specific DNA binding
	LOC102604858	Chr03	transcription factor SPEECHLESS	DNA-binding transcription factor activity
MYB	LOC102584003	Chr07	TSF	cis-regulatory region sequence-specific DNA binding
	LOC102586398	Chr06	myb-related protein Myb4-like	cis-regµlatory region sequence-specific DNA binding
	LOC102589919	Chr07	protein ODORANT1-like	transcription cis-regulatory region binding
	LOC102595712	Chr07	myb-related protein Myb4-like	transcription cis-regµlatory region binding
	LOC102603528	Chr08	transcription factor MYB32-like	cis-regµlatory region sequence-specific DNA binding
	LOC102603892	Chr01	transcription factor MYB35-like	transcription cis-regulatory region binding

NAC	LOC102583210	Chr02	NAC domain-containing protein 86-like	DNA binding
	LOC102583602	Chr03	NAC domain-containing protein 86	DNA binding
	LOC102586730	Chr08	NAC domain-containing protein 83-like	DNA binding
	LOC102592931	Chr06	NAC domain-containing protein 30-like	DNA binding
	LOC102602614	Chr08	NAC domain-containing protein 66-like	Unknown
ERF	LOC102577707	Chr03	CBF3	DNA-binding transcription factor activity
	LOC102590176	Chr12	ethylene-responsive transcription factor WIN1-like	DNA-binding transcription factor activity
	LOC102599677	Chr03	ethylene-responsive transcription factor CRF3-like	unknown
	LOC102605756	Chr10	transcription factor ERF109-like	unknown
LBD	LOC102580843	Chr12	LOB domain-containing protein 4-like	unknown
	LOC102603810	Chr06	LOB domain-containing protein 1-like	unknown
	LOC102606392	Chr05	LOB domain-containing protein 40-like	unknown
HD-ZIP	LOC102583067	Chr02	homeobox-leucine zipper protein ATHB-40-like	DNA-binding transcription factor activity, RNA polymerase II
	LOC102586567	Chr05	homeobox-leucine zipper protein HOX3-like	transcription cis-regulatory region binding
	LOC102594573	Chr10	heat stress transcription factor B-3-like	RNA polymerase II cis-regulatory region sequence-specific DNA

HSF	LOC102601498	Chr04	heat stress transcription factor B-3	DNA-binding transcription factor activity
ZF-HD	LOC102590407	Chr04	zinc finger homeodomain protein 1	DNA-binding transcription factor activity
	LOC102602352	Chr03	zinc-finger homeodomain protein 9-like	transcription cis-regulatory region binding
C2H2	LOC107062282	Chr06	zinc finger protein 1-like	unknown
C3H	LOC102580787	Chr02	zinc finger CCCH domain-containing protein 14-like	mRNA binding
GATA	LOC102596373	Chr02	GATA transcription factor 19-like	zinc ion binding
GRAS	LOC102606215	Chr11	modulation-signaling pathway 2 protein-like	sequence-specific DNA binding
MYB-related	LOC102594971	Chr04	MYB transcription factor	transcription cis-regulatory region binding
TCP	LOC102587457	Chr03	transcription factor TCP19	unknown
YABBY	LOC102604849	Chr01	YABBY1	regulation of shoot apical meristem development
